# Outcomes and trends of peripartum maternal admission to the intensive care unit

**DOI:** 10.1007/s00508-016-1161-z

**Published:** 2017-01-18

**Authors:** Alex Farr, Agnes Lenz-Gebhart, Sabrina Einig, Clemens Ortner, Iris Holzer, Marie Elhenicky, Peter W. Husslein, Rainer Lehner

**Affiliations:** 1 Department of Obstetrics and Gynecology, Division of Obstetrics and Feto-maternal Medicine, Medical University of Vienna, Waehringer Guertel 18–20, 1090 Vienna, Austria; 2 Department of Anesthesia, General Intensive Care Medicine and Pain Therapy, Medical University of Vienna, Vienna, Austria

**Keywords:** Comorbidity, Intensive care unit, Mortality, Near miss, Pregnancy complication

## Abstract

**Background:**

The number of pregnant women with severe comorbidities is increasing. The aim of the present study was to analyze outcomes and determine trends in women who required peripartum admission to the intensive care unit (ICU).

**Methods:**

In this retrospective study, we identified all women who were admitted to the ICU between the second trimester of pregnancy and 6 weeks postpartum. Women with ICU admission between 2011 and 2014 were assigned to the study group, whereas those admitted between 1996 and 2003 were assigned to the historical group. Comorbidities, characteristics, outcomes, and treatment efforts were assessed. Descriptive analysis, Fisher’s exact test, unpaired Student’s *t*-test and one-way ANOVA were performed.

**Results:**

We identified 238 cases, including 135 (56.7%) in the study group and 103 (43.3%) in the historical group. In 83 (34.9%) women, deterioration of the pre-existing medical condition was causal for ICU admission. Overall, preterm delivery and mean gestational age were 81.5% and 31.6 ± 6.2 weeks, respectively. In comparison to the historical group, women of the study group were older (*p* = 0.005), more frequently presented with multiple comorbidities (*p* = 0.003), pre-existing conditions (*p* < 0.001), and congenital heart disease (*p* = 0.012). Moreover, they had a shorter length of stay at the ICU than those of the historical group (*p* = 0.02).

**Conclusions:**

Peripartum ICU admissions are increasing in frequency. As maternal characteristics are changing, adequate risk stratification with multidisciplinary care are essential, and access to intermediate care units would be preferable for patients with short-term admission.

## Introduction

Recent demographic data indicate that the number of women in childbearing age presenting with severe comorbidities is constantly rising [1, 2]. This trend may be a result of the increasing maternal age at the time of the first pregnancy, which was observed in developed countries during the last decades [3, 4]. Another reason for the increasing rate of maternal comorbidities might be the fact that women who underwent modern and innovative medical treatment during the 1980s and 1990s have reached reproductive age and achieved pregnancy [5]. As a consequence, pregnancies of women with pre-existing conditions more often result in peripartum complications and these women frequently require admission to the intensive care unit (ICU) [6]. Indeed, this situation represents a significant challenge for clinicians by causing potentially avoidable complications with increased need for enhanced and multidisciplinary care [7]. Despite the recent achievements of perinatal medicine, increasing maternal illness might also contribute to the steady high rate of preterm delivery (PTD), as well as to direct and indirect maternal morbidity. One of the key reactions to this demographic trend in obstetrics would be an accurate definition of the affected patient population with peripartum ICU admission; however, as the ICU admission criteria are relatively heterogeneous, this has proven to be relatively difficult [8]. Furthermore, data on affected women are mostly outdated and generally unrepresentative for European countries [9].

The objective of the present study was to analyze outcomes and determine trends comparing maternal characteristics and treatment details of women, whose conditions required peripartum ICU admission. In view of recent sociodemographic trends, this information could help to identify women at risk, enable adequate risk stratification, and contribute to the development of a modern approach in perinatal care.

## Patients and methods

The study was approved by the ethics committee of the Medical University of Vienna (reference number 1928/2014, date of approval 2015-01-26), in accordance with the Declaration of Helsinki and the guidelines of good clinical practice. Due to the retrospective character, written informed consent was not obtained. All patient records and data were anonymized and de-identified prior to the analysis. Subsequently, we retrospectively collected the data of all consecutive women who required peripartum admission to the ICU at the Medical University of Vienna. Eligible women were in the second or third trimester of pregnancy or up to 6 weeks postpartum at the time of ICU admission. Those who were admitted to a psychiatric ICU were not eligible for study inclusion. In order to determine trends in maternal characteristics, we differentiated between women who were treated between 1 January 2011 and 31 December 2014 (defined as the study group), and those treated between 1 January 1996 and 31 December 2003 (defined as the historical group). Given the fact that no data were available for the years 2004–2010, time intervals were chosen for comparable case numbers in the study groups.

The study was undertaken at the Medical University of Vienna, Vienna General Hospital, which is a 2200 bed tertiary referral center, providing medical attendance for approximately 99,000 inpatients and 500,000 outpatients per year. The Department of Obstetrics and Gynecology is specialized in high-risk obstetrics and serves up to 3000 deliveries per year. Referrals are received from the whole of Austria and Central Eastern Europe. Women requiring intensive care treatment are admitted to in-house intensive care units that are operated by the Department of Anesthesia, General Intensive Care Medicine and Pain Therapy.

We identified eligible patients by collaborating with the hospital controlling, providing the departmental list of all ICU admissions. Patient charts were electronically reviewed using the PIA Fetal Database, version 5.6.16.917 (General Electric Company, GE Viewpoint, Wessling, Germany) and the hospital information system (SAP SE, Walldorf, Germany). Medical records of women with incomplete or inconclusive data were manually retrieved and reviewed. Extracted information included patient demographics, individual comorbidities, pre-existing medical conditions, pregnancy and delivery details, maternal and neonatal outcome parameters, as well as treatment details during ICU hospitalization, and the intensive care severity of illness score acute physiology and chronic health evaluation (APACHE II) [10]. According to the uniform criteria of the World Health Organization (WHO), women were defined as “near miss” in cases where they nearly died, but survived a complication that occurred during pregnancy, childbirth or within 42 days postpartum [6]. Gestational age was described as weeks plus days after the last normal menstrual period. Pre-existing or pregnancy-induced hypertension was defined as blood pressure higher than 140/90 mm Hg, diagnosed before or after 20 weeks of gestation, measured on 2 separate occasions more than 6 hours apart. Preterm delivery (PTD) was defined as delivery of a neonate prior to 37 + 0 gestational weeks. Delivery data included patients with antepartum and postpartum ICU admission. Stillbirth was defined as the term or preterm delivery of a neonate with a birth weight of ≥500 g and an Apgar score of 0/0/0. Demographic information is summarized and displayed using descriptive statistics. Discrete data are presented as *N* (%). Continuous data are given as mean ± standard deviation (SD). In case of skewed data distribution, data are given as median and interquartile range (IQR, range from the 25th to the 75th percentile). Fisher’s exact test was used to compare groups of categorical data. Continuous data were compared using the unpaired Student’s *t-*test or one-way analysis of the variance. A two-sided *p* value <0.05 was considered statistically significant. Statistical calculations were performed using SPSS Statistics, version 23.0 (IBM SPSS, NY).

## Results

### Patient characteristics

Comprehensive research led to the identification of 238 eligible cases. The mean age of the women at the time of delivery was 30.6 ± 6.3 years. Out of the study population, 138 (58%) women were primiparous and 100 (42%) multiparous. Rates of multiple pregnancy and assisted reproduction were 9.7% and 6.8%, respectively. A total of 83 (34.9%) women presented with pre-existing medical conditions during pregnancy. Multiple comorbidities were reported in 49 (20.6%) women. Pre-existing medical conditions included hypertensive disorders in 15 (6.3%), diabetes in 12 (5%), bronchial asthma in 4 (1.7%), congenital heart disease (CHD) in 15 (6.3%), rheumatic disease in 10 (4.2%), opioid dependence in 8 (3.4%), and other comorbidities, such as previous organ transplantation, clotting disorders, neurologic, renal or malignant diseases in 19 (8%) women. Patient characteristics are summarized in Table 1.

**Table 1:** Baseline variables of 238 study patients with peripartal ICU admission

Variable	Peripartal ICU admission		All	Test
	Study group	Historical group		
	Mean ± SD *N* (%)	Mean ± SD *N* (%)	Mean ± SD *N* (%)	*p* value
Participants	135 (56.7)	103 (43.3)	238 (100)	n. t.
Age at delivery (years)	31.6 ± 6.4	29.3 ± 5.8	30.6 ± 6.3	0.005
Parity				
Primiparae	78 (57.8)	60 (58.3)	138 (58)	n. s.
Multiparae	57 (42.2)	43 (41.7)	100 (42)	n. s.
Multiple pregnancy^a^	17 (12.6)	6 (5.8)	23 (9.7)	n. s.
Assisted reproduction	16 (6.8)	n/a	n/a	n. t.
Multiple comorbidities^b^	37 (27.4)	12 (11.7)	49 (20.6)	0.003
Pre-existing condition				
Yes	63 (46.6)	20 (19.4)	83 (34.9)	<0.001
No	72 (53.4)	83 (80.6)	155 (65.1)	
Pre-existing condition (detail)				
Hypertension	12 (8.9)	3 (2.9)	15 (6.3)	n. s.
Diabetes	8 (5.9)	4 (3.9)	12 (5)	n. s.
Bronchial asthma	3 (2.2)	1 (1)	4 (1.7)	n. s.
Congenital heart disease	13 (9.6)	2 (1.9)	15 (6.3)	0.012
Rheumatic disease	7 (5.2)	3 (2.9)	10 (4.2)	n. s.
Opioid dependence	7 (5.2)	1 (1)	8 (3.4)	n. t.
Others^c^	13 (9.6)	6 (5.8)	19 (8)	n. s.

*N* number, *SD* standard deviation, *n/a* no data available, *n. s.* not significant, *n. t.* not tested

^a^ incl. 2 triplets in the study group, ^b^ defined as >1 pre-existing condition, ^c^ incl. previous organ transplantations, clotting disorders, neurologic, renal and/or malignant diseases

### Obstetric outcomes

Women who required peripartum ICU admission delivered at a mean of 31.6 ± 6.2 gestational weeks, corresponding to a PTD rate of 81.5%. The live birth and cesarean section rates were 86.6% and 84.5%, respectively. The majority of women (77.7%) were admitted to the ICU postpartum and 70.2% underwent antenatal care at our center. The mean birthweight at delivery in the observed population was 2021 ± 963 g. Indications for ICU admission included deterioration of the pre-existing maternal condition or pregnancy-related complications, such as pregnancy-induced hypertension, preeclampsia and HELLP (hemolysis, elevated liver enzymes and low platelets) syndrome in 76 (31.9%) women, postpartum hemorrhage (PPH) in 38 (16%), intracerebral bleeding (ICB) in 15 (6.3%), infection or sepsis in 17 (7.1%), thrombosis or embolism in 10 (4.2%) and respiratory failure in 8 (3.4%) women. A total of 30 (12.6%) women underwent postpartum hysterectomy. Table 2 shows the detailed obstetric outcomes of the study population.

**Table 2:** Obstetric outcomes of 238 study patients with peripartum ICU admission

Variable	Category	Study group (*N* = 135)	Historical group (*N* = 103)	All (*N* = 238)	Test
	Unit	Mean ± SD % (*N*)	Mean ± SD % (*N*)	Mean ± SD % (*N*)	*p *value
Pregnancy outcome	Live birth Stillbirth	123 (91.1) 12 (8.9)	83 (80.6) 20 (19.4)	206 (86.6) 32 (13.4)	0.018
Prematurity	Preterm delivery No preterm delivery	119 (88.2) 16 (11.8)	75 (72.8) 28 (27.2)	194 (81.5) 44 (18.5)	0.003
Antenatal referral	Antenatal referral No antenatal referral	31 (23) 104 (77)	40 (38.8) 63 (61.2)	71 (29.8) 167 (70.2)	0.008
Mode of delivery	Vaginal delivery^a^ Cesarean section Instrumental delivery	9 (6.7) 124 (91.9) 2 (1.4)	23 (22.3) 77 (74.8) 3 (2.9)	32 (13.4) 201 (84.5) 5 (2.1)	0.001
Gestational age at delivery	Weeks	31.5 ± 5.5	31.8 ± 7.1	31.6 ± 6.2	n. s.
	<24+0 24+0–27+6 28+0–33+6 34+0–36+6 ≥37+0	8 (5.9) 15 (11.1) 48 (35.6) 48 (35.6) 16 (11.8)	11 (10.7) 12 (11.7) 20 (19.4) 32 (31) 28 (27.2)	19 (8) 27 (11.3) 68 (28.6) 80 (33.6) 44 (18.5)	n. t.
Birthweight^b^	Grams	1897 ± 880	2215 ± 1055	2021 ± 963	0.019
	<500 g 500–999 g 1000–1499 g 1500–2499 g ≥2500 g	6/154 (3.9) 25/154 (16.2) 24/154 (15.6) 46/154 (29.9) 42/154 (27.3)	6/109 (5.5) 11/109 (10.1) 7/109 (6.4) 21/109 (19.3) 46/109 (42.2)	12/263 (4.6) 36/263 (13.7) 31/263 (11.8) 67/263 (25.5) 88/263 (33.5)	n. t.
Time of ICU admission	Antepartum Postpartum	18 (13.3) 117 (86.7)	35 (34) 68 (66)	53 (22.3) 185 (77.7)	<0.001
Hysterectomy	Hysterectomy No hysterectomy	13 (9.6) 122 (90.4)	17 (16.5) 86 (83.5)	30 (12.6) 208 (87.4)	n. s.

*N* number, *SD* standard deviation, *n. s.* not significant, *n. t.* not tested, *ICU* intensive care unit

^a^including breech delivery, ^b^data of *N* = 11 (study)/*N* = 18 (historical)/*N* = 29 (all) not available (neonates are denominators)

### Treatment details

The median length of stay at the ICU was 3 (IQR 1–5) days, whereas the median total duration of hospitalization was 13 (IQR 9–20) days. A total of 81 (34%) women underwent mechanical ventilation. General anesthesia, spinal anesthesia and epidural anesthesia were used in 104 (43.7%), 101 (42.4%) and 13 (5.5%) women, respectively. Monitoring was either non-invasive (60%), with invasive blood pressure (30.3%) or with pulmonary artery pressure (9.7%). A total of 12 (5%) women died after ICU admission, whereas 119 (50%) were near misses and 107 (45%) recovered. Out of 101 (42.4%) women who received blood transfusions, 60 (25.2%) underwent massive transfusion with more than 4 erythrocyte concentrates. Interventions and treatment details are listed in Table 3.

**Table 3:** Treatment details of 238 study patients with peripartum ICU admission

Variable	Category	Study group (*N* = 135)	Historical group (*N* = 103)	All (*N* = 238)	Test
	Unit	Median (IQR) % (*N*)	Median (IQR) % (*N*)	Median (IQR) % (*N*)	*p* value
Obstetric anesthesia	Spinal Epidural General None	77 (57) 7 (5.2) 48 (35.6) 3 (2.2)	24 (23.3) 6 (5.8) 56 (54.4) 17 (16.5)	101 (42.4) 13 (5.5) 104 (43.7) 20 (8.4)	<0.001
Monitoring	Non-invasive Invasive blood pressure Pulmonary artery pressure	106 (78.5) 21 (15.6) 8 (5.9)	37 (35.9) 51 (49.5) 15 (14.6)	143 (60) 72 (30.3) 23 (9.7)	<0.001
Ventilation^b^	Days	3 (1–10)	1 (1–10)	2 (1–10)	n. s.
	Ventilation >7 days Ventilation <7 days No ventilation	9 (6.7) 23 (17) 103 (76.3)	16 (15.5) 33 (32) 54 (52.5)	25 (10.5) 56 (23.5) 157 (66)	n. t.
ICU outcome	Recovered Near miss Death	69 (51.2) 60 (44.4) 6 (4.4)	38 (36.9) 59 (57.3) 6 (5.8)	107 (45) 119 (50) 12 (5)	n. s.
Intervention	EC^a^ PC FFP Antibiotics Antihypertensive Catecholamine LMWH intravenously HF/HD CPR ECMO	45 (33.3) 11 (8.1) 15 (11.1) 85 (63) 63 (46.7) 21 (15.6) 8 (5.9) 2 (1.5) 7 (5.2) 6 (4.4)	56 (54.4) 21 (20.4) 3 (2.9) 80 (77.7) 25 (24.3) 28 (27.2) 11 (10.7) 12 (11.7) 8 (7.8) 4 (3.9)	101 (42.4) 32 (13.4) 18 (7.6) 165 (69.3) 88 (37) 49 (20.6) 19 (8) 14 (5.9) 15 (6.3) 10 (4.2)	0.001 0.006 0.018 0.015 0.002 0.028 n. s. 0.001 n. s. n. s.
Primary organ failure	Cardiovascular/respiratory Renal Cerebral Hepatic Not specified	74 (54.8) 10 (7.4) 5 (3.7) 13 (9.6) 33 (24.5)	77 (74.8) 4 (3.9) 2 (1.9) 4 (3.9) 16 (15.5)	151 (63.4) 14 (5.9) 7 (2.9) 17 (7.2) 49 (20.6)	0.045
Length of stay	Days at ICU Total duration	3 (1–4) 13 (10–18)	3 (1–7) 12 (9–22)	3 (1–5) 13 (9–20)	0.02
					n. s.

*N* number, *SD* standard deviation, *IQR* interquartile range, *n/a* no data available, *n. s.* not significant, *n. t.* not tested, *EC* erythrocyte concentrate, *PC* platelet concentrate, *FFP* fresh frozen plasma, *LMWH* low molecular weight heparin, *HF/HD* hemofiltration/hemodialysis, *ECMO* extracorporeal membrane oxygenation, *CPR* cardiopulmonary resuscitation, *ICU* intensive care unit

^a^including massive transfusion in *N* = 17 (study)/*N* = 43 (historical)/*N* = 60 (all), ^b^apart from ventilation during cesarean section

### Trends and changes

**Fig. 1:**
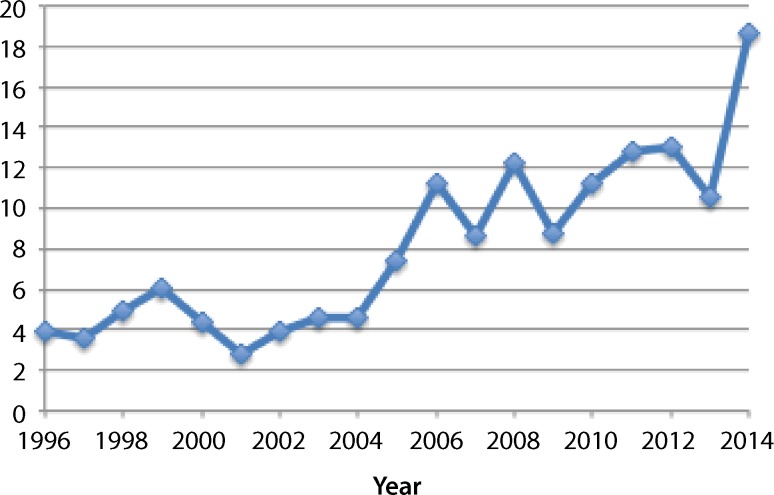
ICU admissions per 1000 deliveries from 1996–2014

Out of 37,236 deliveries during the analyzed time period, the mean number of patients admitted to the ICU per year increased from 14.9 ± 3.9 in the historical group to 33.8 ± 12.3 in the study group (*p* = 0.05). There was an increasing trend of ICU admissions per 1000 deliveries per year, as demonstrated in Fig. 1. Mean admission rates in the historical and study group were 0.4 ± 0.1% and 1.4 ± 0.4%, respectively (*p* = 0.01). Compared to the historical group, women of the study group were older (*p* = 0.005), and they more frequently presented to the ICU with pre-existing medical conditions (*p* < 0.001). No significant differences were found regarding the rate of multiple pregnancy. CHD was more frequently found in the study group than in the historical group (*p* = 0.012). Evaluation of obstetric outcomes showed higher live birth (*p* = 0.018) and PTD rates (*p* = 0.003) in women of the study group. Antenatal referrals from other hospitals were performed more often in the study group (*p* = 0.008), as well as cesarean sections (*p* = 0.001). The mean birthweight was 1897 ± 880 g in the study group and 2215 ± 1055 g in the historical group (*p* = 0.019). No significant differences were found regarding hysterectomy (Table 2). General anesthesia was less frequently performed in the study group than in the historical group (35.6% versus 54.4%; *p* < 0.001). Invasive blood pressure and pulmonary artery monitoring was less frequently used in the study group than in the historical group (*p* < 0.001). Maternal mortality rates were 4.4% in the study group and 5.8% and the historical group, which was not significantly different. Antihypertensive medication was more often administered in the study group, whereas blood transfusions, platelet concentrates, antibiotics, catecholamine, and hemodialysis were more often administered in the historical group. The median APACHE II score was 7 (IQR 4–9). The APACHE II score was not available for the historical group.

## Discussion

The present study provides one of the largest datasets available on obstetric ICU admissions in Europe. We were able to identify trends of changing maternal characteristics, such as an increase of maternal age and comorbidities, as well as a shorter length of stay at the ICU during recent times.

Pollock et al. [9] reported an overall incidence of obstetric ICU admission of 2.7 per 1000 deliveries, equating to 1 admission per 370 deliveries. In our study, we observed an admission rate of 6.4 per 1000 deliveries, corresponding to 1 admission per 156 deliveries. Compared to other studies, this number is rather high, but should be interpreted within the tertiary setting of our study. One of our main findings is the increasing maternal age of women with ICU admission, which is also higher than the overall obstetric population in Austria [11]. Although previous studies evaluating the association of maternal age and ICU admission have reported conflicting results, it can be stated that women of higher age frequently suffer from hypertensive disorders and various diseases that might be related to ICU admission [12–14].

In our study, we found that ICU admission was frequently associated with the presence and deterioration of pre-existing medical conditions. This is in accordance with the data of Neligan and Laffey [15]. In addition, missing pregnancy care has been identified as a risk factor for peripartum ICU admission, which we could not analyze in our dataset [12]. Hazelgrove et al. [16] evaluated data of women who were admitted to the ICU during the 1990s, reporting pre-existing conditions in 23% of the observed patients. The trend of increasing rates of pre-existing maternal conditions in obstetrics reflects a demographic trend that is caused by changing maternal characteristics, such as increasing age and also by improved obstetric care during recent years. The increasing number of women with CHD, who became pregnant and were admitted to the ICU, suggests that they underwent the novel cardiac surgery techniques and treatments, which were introduced during the past decade. This might have led them reach reproductive age and achieve pregnancy. In the light of these trends, the 5% mortality rate, which we observed in our study, might be considered as appropriate in comparison to the literature [17]. Most of the 12 women who died were primiparae with at least one pre-existing condition, experiencing either serious pregnancy-related complications or deterioration of a pre-existing disease. Mortality due to PPH, which is still the leading cause of maternal death worldwide, was not observed in our cohort. This might be a result of effective treatment options and standardized PPH management [18, 19].

What further aroused our interest was that we observed that the majority of patients were admitted to the ICU for monitoring and recovery. In fact, many women in the study group required neither ventilation nor invasive monitoring. As evidenced by the significant reduction in the use of invasive blood pressure monitoring, catecholamines and hemofiltration, invasive intensive care seems to be required for a few selected patients. This suggests that many women who are nowadays admitted to the ICU could also benefit from an obstetric intermediate care unit (IMCU). Trauma units or burn intensive care units could serve as models for a bidisciplinary treatment approach at the obstetric IMCU, which could be managed under the leadership of specially trained obstetricians in close collaboration with anesthesiologists and intensive care specialists. For women who need to be admitted for postpartum monitoring and recovery, this approach could save healthcare costs. We need to admit that our study has several limitations. The study was conducted in a high-risk obstetric setting with comprehensive access to intensive care. Consequently, the results should be interpreted within this setting. Moreover, missing data on the APACHE score seems to detract our results. However, this score is known to overestimate mortality rates in obstetric populations [20]. Since the vast majority of published studies on this topic used a simple observational design, we decided to perform an historical cohort study. This allowed us to identify trends, which we found were beneficial for this paper. Nevertheless, some of the observed differences might reflect changing obstetric practice. There might have been changes in the admission policy that accounted for increasing ICU admission rates. The increasing number of women, who were admitted to the ICU, might also arise from an increase in population or from the introduction of new diagnostic and treatment modalities. Essentially, significant advances in critical care and blood management were made throughout the study period. As a result, prospective assessment, electronic documentation, and multicenter design may enhance the quality of subsequent studies.

In conclusion, our data indicate that peripartum ICU admissions are increasing in frequency with a remarkable number of affected women, who suffer from serious pre-existing conditions. Nowadays, women who require peripartum ICU admission are older and commonly present with multiple comorbidities. In modern obstetrical care, a multidisciplinary approach with adequate risk stratification prior to pregnancy, early registration for delivery, and continuous pregnancy care is needed to improve peripartum outcomes and react to the sociodemographic trends of recent years. The shorter length of stay at the ICU suggests an increasing need for the broad availability of obstetric intermediate care units, which could be suitable for non-ventilated women, who require short-term postpartum ICU admission.
